# Diagnostic Yield of the Light Blue Crest Sign in Gastric Intestinal Metaplasia: A Meta-Analysis

**DOI:** 10.1371/journal.pone.0092874

**Published:** 2014-03-21

**Authors:** Lei Wang, Wei Huang, Jing Du, Youwei Chen, Jianmin Yang

**Affiliations:** Department of Gastroenterology, Zhejiang Provincial People's Hospital, Hangzhou, Zhejiang, China; University Hospital Llandough, United Kingdom

## Abstract

**Background:**

The diagnostic yield of light blue crest(LBC) sign, which was observed by narrow band imaging with magnification endoscopy(NBI-ME), in detecting gastric intestinal metaplasia(IM) has shown variable results.

**Objective:**

We aimed to assess the diagnostic value of LBC under NBI-ME for detecting gastric IM.

**Methods:**

We performed a literature search of the Medline/PubMed, Embase, Web of Science, Science Direct and the Cochrane Library Databases; and a meta-analysis of pooled sensitivity, specificity, positive likelihood ratio, negative likelihood ratio, and SROC area under the curve, using fixed- and random-effects models, for the accuracy of LBC-based IM diagnosis.

**Results:**

We initially included 4 articles, but excluded 1 article to counter significant heterogeneity. When pooled, the remaining 3 articles, which included 247 patients with 721 lesions, showed the following patterns in IM diagnosis: sensitivity: 0.90 (95% confidence interval [CI] 0.86–0.92); specificity: 0.90 (95% CI 0.86–0.93), positive likelihood ratio: 8.98 (95% CI 6.42–12.58), negative likelihood ratio: 0.12 (95% CI 0.09–0.16), and SROC area under the curve: 0.9560.

**Limitations:**

As the studies varied by their definitions for positive LBC, endoscopy types, biopsy protocols, race of patient cohort, and physicians' proficiency, some sample sizes were limited so that subgroup analyses could not be performed.

**Conclusion:**

We concluded that observing LBC under NBI-ME is an accurate and precise means of diagnosing gastric IM.

## Introduction

Gastric intestinal metaplasia (IM) is regarded as a precancerous lesion [1], accurate surveillance of which could lead to early detection and treatment before further progression, thus preventing gastric cancer and improving patient survival [2]. Hence, diagnosis and surveillance of IM by endoscopy is of great value. As distribution of IM is patchy and is not distinctly visible by routine white-light endoscopy, use of the random biopsy technique is subject to sampling error [3], [4]. Narrow band imaging (NBI) is a real-time, on-demand endoscopic imaging technique designed to enhance visualization of the vascular network and surface texture of the mucosa by use of narrower bands of blue and green filters, which are different from conventional red-green-blue filters [5], [6]. The light blue crest (LBC), a blue-whitish patchy area observable with NBI-magnification endoscopy (ME) on the gastric epithelial surface, may have a distinctive endoscopic diagnostic appearance of IM [3].

This meta-analysis aimed to assess the diagnostic accuracy, sensitivity, and specificity of LBC under NBI-ME in diagnosing gastric IM.

## Materials and Methods

### Search strategy

Database searches were performed up to May 2013 in Medline/PubMed, Embase, Web of Science, Science Direct and the Cochrane Library, using two alternative search terms: “Narrow Band Imaging” and “intestinal metaplasia” and “diagnosis”; or “intestinal metaplasia” and “light blue crest”. References in available articles were also reviewed.

### Selection of studies

Studies were selected according to the inclusion criteria: (1) aim of clarifying the accuracy of LBC in diagnosing gastric IM; (2) use of the Updated Sydney System Criteria or Chinese Consensus for the Diagnosis of Chronic Gastritis as pathological diagnostic criteria; (3) use of NBI-ME in all subjects, followed by the pathological examination; (4) use of “with or without LBC” as the standard to diagnose gastric IM under endoscopy; (5) recruitment of non-specific population for the study; (6) calculations for true or false-positive value, and true or false-negative value by directly or indirectly acquired LBC in gastric IM. We excluded studies (a) with specific populations or restricted age, gender or etiology; (b) reviews, lectures and comments; or (c) with no definite diagnostic criteria or diagnostic criteria that were incompatible with those in our study.

### Diagnostic criteria

#### Diagnostic criteria for LBC and IM under NBI-ME

**Figure 1:**
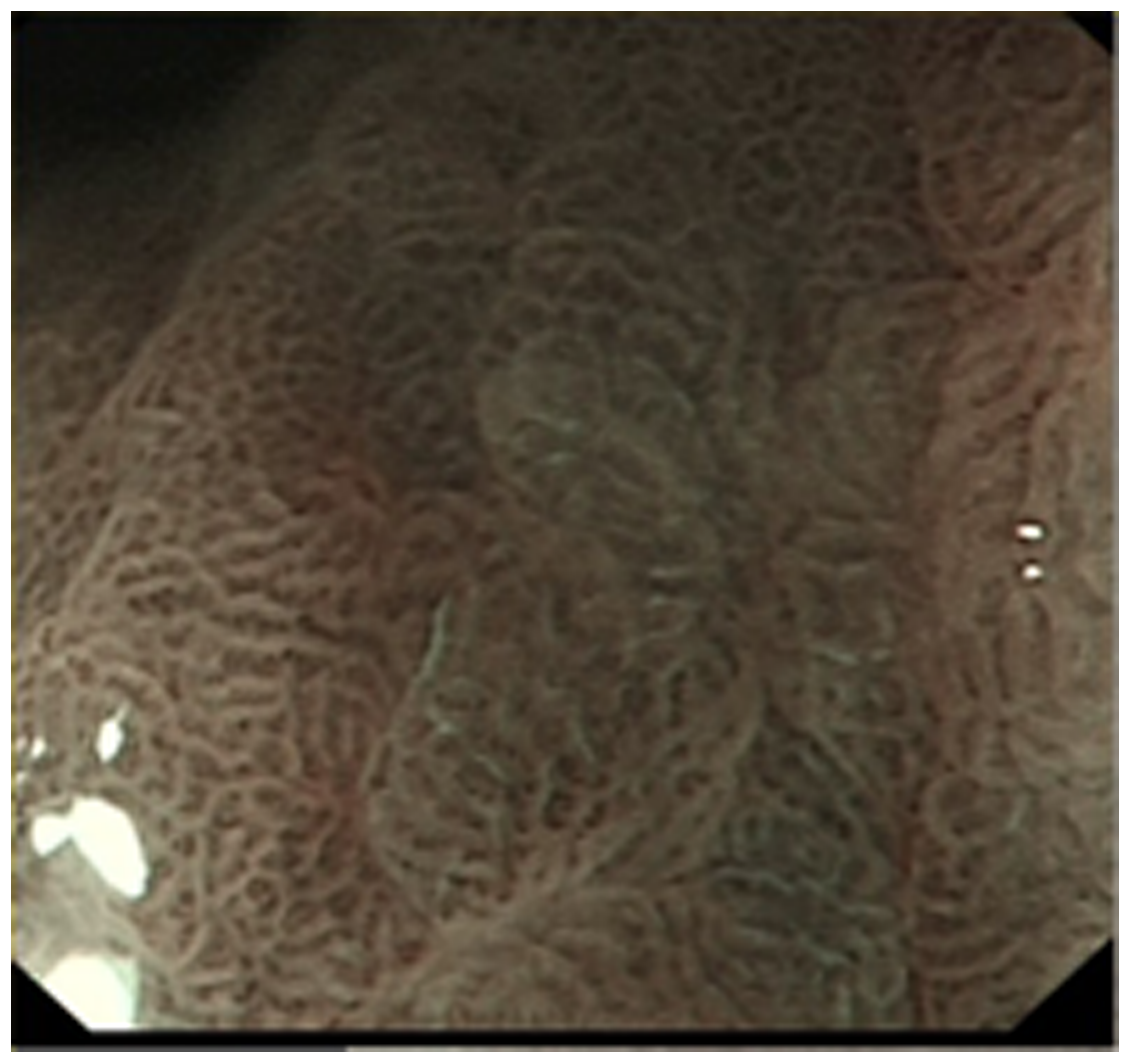
Light blue crest sign. Light blue crest (LBC) appears as blue-whit lines visible on the epithelial surface under narrow band imaging with magnification endoscopy (NBI-ME) (original photo, Olympus GIF-H260Z, under 80×magnification).

LBC was defined as a fine, blue-white line on crests of epithelial surfaces/gyri, seen under NBI-ME [3] (Figure 1). IM was diagnosed by the “with or without LBC” standard. Patients were classed as IM^+^ if LBC was seen in any of the image fields, and otherwise as IM^−^ [3].

#### Pathological criteria for IM

IM was diagnosed according to the Updated Sydney System criteria. The diagnostic criteria of chronic gastritis in China are consistent with the Updated Sydney System criteria. Studies with these criteria were also included [7], [8].

### Data extraction and Assessment of study quality

All included studies were assessed and data were extracted using a predefined data extraction form. The following variables were assessed: author, year and country of publication and endoscope type used. True positives, false positives, false negatives and true negatives were extracted using the histological findings as gold standard. The data were extracted both on a ‘per-patient’ and a ‘per-lesion’ basis wherever available.

The quality of the studies was assessed using the Quality Assessment of Diagnostic Accuracy Studies-2 (QUADAS-2) tool (Table 1). The tool consists of four key domains that cover patient selection, index test, reference standard, and flow of patients through the study and timing of the index test(s) and reference standard (“flow and timing”). The tool is completed in four phases: (1) statement of the review question; (2) development of review-specific guidance; (3) review of the published flow diagram for the primary study or construction of a flow diagram if none is reported; (4) judgment of bias and applicability. Each domain is assessed in terms of the risk of bias, and the first three are also assessed in terms of concerns regarding applicability. To help reach a judgment on the risk of bias, signaling questions are included. Risk of bias is judged as “low”, “high”, or “unclear”. If all signaling questions for a domain are answered in the affirmative, then risk of bias can be judged “low”. If any signaling question is answered in the negative, this flags the potential for bias. The “unclear” category should be used only when insufficient data are reported to permit a judgment. Review authors were asked to record information on which the judgment of applicability is made and then to rate their concern that the study does not match the review question. Concerns regarding applicability are rated as “low”, “high” or “unclear” [9]. The literature was searched, and evaluated by two independent investigators (Lei Wang and Wei Huang); consensus was obtained after consultation.

**Table 1:** Original Table of Quality Assessment of Diagnostic Accuracy Studies-2 (QUADAS-2) tool [9].

Domain	Patient Selection	Index Test	Reference Standard	Flow And Timing
Description	Describe methods of patient selection	Describe the index test and how it was conducted and interpreted	Describe the reference standard and how it was conducted and interpreted	Describe any patients who did not receive the index test(s) and/or reference standard or who were excluded from the 2×2 table (refer to flow diagram): Describe the time interval and any interventions between index test(s) and reference standard
	Describe included patients (prior testing, presentation, intended use of index test and setting)			
Signaling questions (yes/no/unclear)	Was a consecutive or random sample of patients enrolled?	Were the index test results interpreted without knowledge of the results of the reference standard?	Is the reference standard likely to correctly classify the target condition?	Did all patients receive a reference standard?
	Was a case-control design avoided?	If a threshold was used, was it pre-specified?	Were the reference standard results interpreted without knowledge of the results of the index test?	Did all patients receive the same reference standard?
	Did the study avoid inappropriate exclusions?			Were all patients included in the analysis?
Risk of bias: (high/low/unclear)	Could the selection of patients have introduced bias?	Could the conduct or interpretation of the index test have introduced bias?	Could the reference standard, its conduct, or its interpretation have introduced bias?	Could the patient flow have introduced bias?
Concerns regarding applicability: (high/low/unclear)	Are there concerns that the review question is not applicable to the included patients ?	Are there concerns that the review question is not applicable to the index test, its conduct, or its interpretation?	Are there concerns that the the review question is not applicable to the target condition as defined by the reference standard?	

### Data analysis

Meta-DiSc (version 1.4) software was used to estimate heterogeneity due to threshold variation with Spearman correlation coefficient. Cochran's Q and inconsistency (*I* ^2^) for diagnostic odds ratio (DOR) were measured to estimate heterogeneity due to a non-threshold effect. When heterogeneity was present, a random-effects model was used for meta-analysis; otherwise, a fixed-effects model was used. Pooled sensitivity, specificity, positive likelihood ratio, and negative likelihood ratio were calculated. Meta-DiSc software was used to delineate a symmetric receiver operator characteristic (SROC) curve from which the area under curve (AUC) was then calculated.

## Results

### Selection of studies

**Figure 2:**
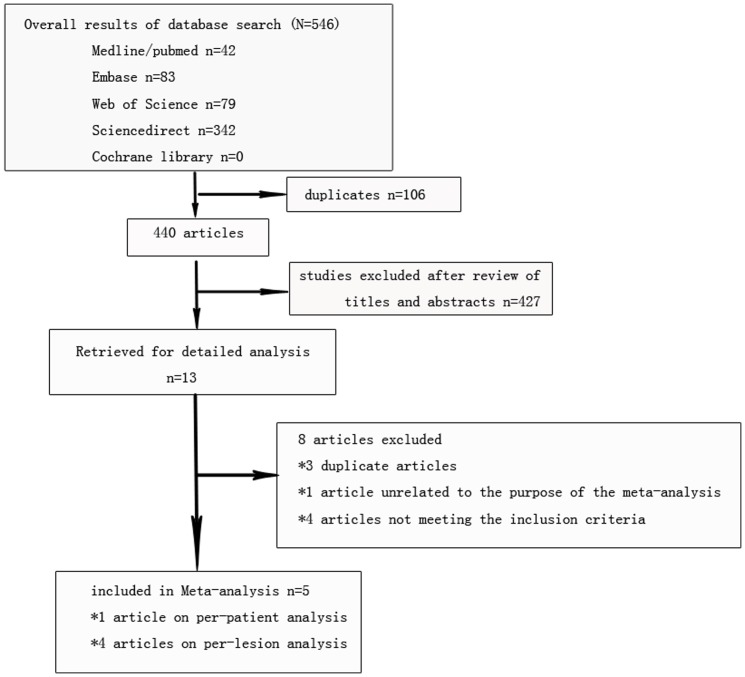
Study selection flow diagram.

The study selection process is summarized in Figure 2. Of the initial 546 studies that were identified in the literature, 106 duplicates were excluded and 427 were excluded because of unrelated titles and abstracts. In the remaining 13 articles that were included in this analysis [3], [10]–[21], four were meeting summaries [13], [18]–[20], and the other nine were published in full text. Some meeting summaries with similar author lists, publishing times and contexts to published papers were also excluded [13], [17], [18], [20], [21]. The remaining 10 articles were thoroughly reviewed [3], [10]–[12], [14]–[17], [19], [21]. One article unrelated to this study was excluded [12]. Four articles that did not meet the inclusion criteria were also excluded (the definition of positive LBC were not consistent with ours in two articles [14], [16], and ME was not performed in two articles [15], [19]). Thus, five articles were finally included for further analysis [3], [10], [11], [17], [21]. Four of these analyzed for data on discriminating IM lesions by per-lesion basis [3], [10], [11], [17], and one study on per-patient basis [21]. One study used a learning set followed by validation on a different cohort [3]. We did not include the learning set in the data analysis. In one study, patients were followed-up the following year [17], but only the initial examination data were included for the further meta-analysis. Details of the included studies are summarized in Table 2.

**Table 2:** Characteristics of studies included in meta-analysis of accuracy of light blue crest signto diagnose gastric intestinal metaplasia.

Authors year, reference	country	Mean age, yrs (range)	Patient (n) (Male/Female)	Total lesions examined (n)	Endoscopy type
Uedo N 2006 [3]	Japan	?	107(?/?)	219	Olympus GIF−Q240Z
Zhou Y 2012 [10]	China	?(42–76)	60(25/35)	314	Olympus GIF−Q260Z
Bian-ying Liu 2009 [11]	China	50.9(33–74)	80(53/27)	188	Olympus GIF−Q240Z
Rerknimitr R 2011 [17]	Thailand	59.9±11.5 (27–80)	38(20/18)	228	Olympus GIF−Q160Z
Savarino E 2013 [21]	Italy	67±12	100(42/58)	500	Olympus GIF−Q160Z

### Evaluation of the study quality

The quality of the included studies was evaluated as shown in Table 3. The five studies represented are of high quality.

**Table 3:** Application of the Quality Assessment of Diagnostic Accuracy Studies-2 (QUADAS-2) tool to the five studies included in the meta-analysis.

Authors			Risk of bias		Applicability Concerns		
	Patient selection	Index test	Reference standard	Flow and timing	Patient selection	Index test	Reference standard
Uedo N [3]	↑	↑	↑	↑	↑	↑	↑
Zhou Y [10]	↑	↑	↑	↑	↑	↑	↑
Bian-ying Liu [11]	↑	↑	↑	↑	↑	↑	↑
Rerknimit R [17]	↑	↑	↑	↑	↑	↑	↑
Savarino E [21]	↑	↑	↑	↑	↑	↑	↑

↑ low risk of bias.

### Meta-analysis

**Figure 3:**
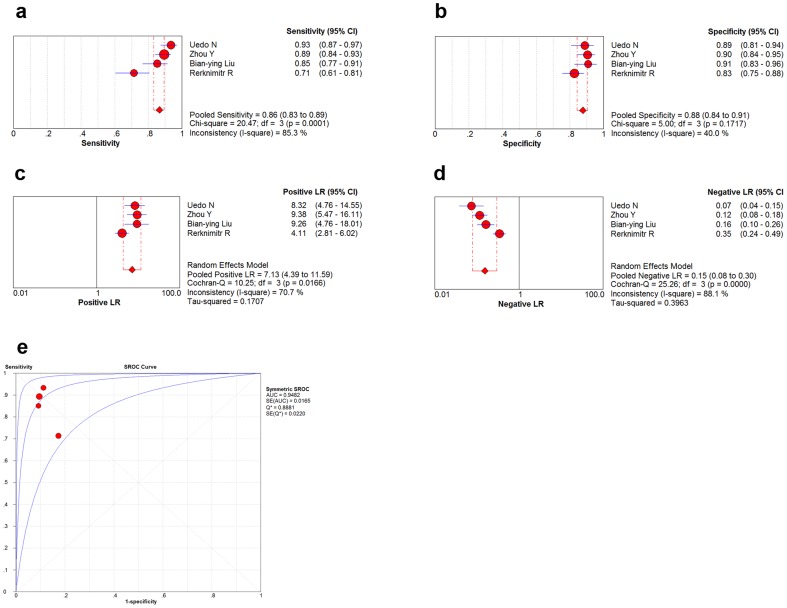
Results of per-lesion analysis of four studies. Per-lesion analysis of diagnostic performance of the light blue crest (LBC) sign under narrow band imaging with magnification endoscopy (NBI-ME) to diagnose gastric intestinal metaplasia(IM): (**a**) pooled sensitivity; (**b**) pooled specificity; (**c**) pooled positive likelihood ratio; (**d**) pooled negtive likelihood ratio; (**e**) symmetric receiver operator curve characteristic (SROC) curve and area under curve(AUC).

Finally, four studies with per-lesion data were included for meta-analysis (a total of 949 samples from 285 patients) [3], [10], [11], [17]. When heterogeneity of these four articles was tested, the Spearman correlation coefficient was ^−^0.200 (*p* = 0.800), which indicates a lack of definite threshold-effect-induced heterogeneity. The Cochran's Q and *I* ^2^ for DOR were 21.99 (*p* = 0.0001) and 86.4% respectively, which indicates non-threshold-effect-induced heterogeneity. A random-effects model was used for meta-analysis. After analysis with the random-effects model, the pooled sensitivity was 0.86 (95% CI: 0.83–0.89), specificity was 0.88 (95% CI: 0.84–0.91), the positive likelihood ratio was 7.131 (95% CI: 4.39–11.59) and negative likelihood ratio was 0.15(95% CI: 0.08–0.30) (Figure 3a–3d). SROC analysis showed the AUC was 0.9482 (Figure 3e).

**Figure 4:**
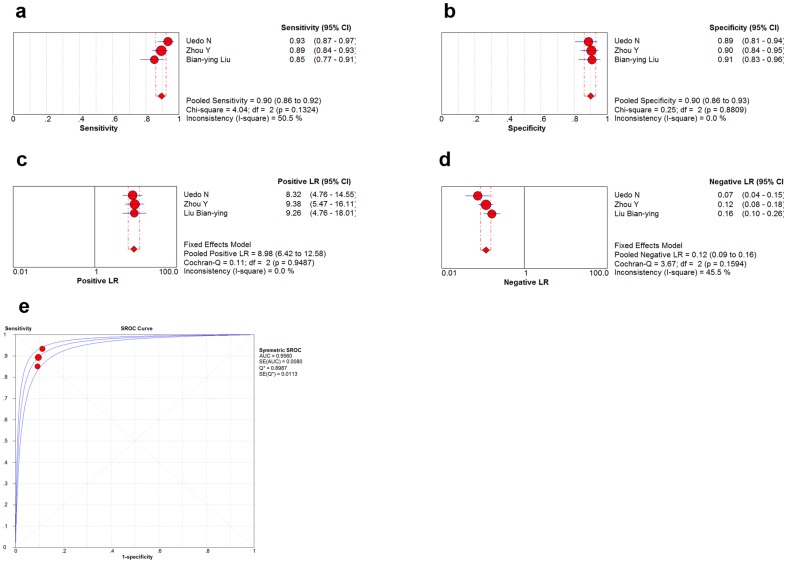
Results of per-lesion analysis of three studies. Per-lesion analysis of diagnostic performance of the light blue crest (LBC) sign under narrow band imaging with magnification endoscopy (NBI-ME) to diagnose gastric intestinal metaplasia(IM): (**a**) pooled sensitivity; (**b**) pooled specificity; (**c**) pooled positive likelihood ratio; (**d**) pooled negtive likelihood ratio; (**e**) symmetric receiver operator curve characteristic (SROC) curve and area under curve(AUC).

The SROC curve showed that one study [17] was presented with bias when compared with other studies [3], [10], [11]. After removing this study, the Cochran's Q and *I* ^2^ for DOR were 1.02 (*p* = 0.5996) and 0% respectively, indicating that there was no heterogeneity among the remaining studies (721 samples from 247 patients). After analysis with a fixed-effects model, pooled sensitivity was 0.90 (95%CI: 0.86–0.92), specificity was 0.90 (95%CI: 0.86–0.93), positive likelihood ratio was 8.98 (95%CI: 6.42–12.58) and negative likelihood ratio was 0.12 (95%CI: 0.09–0.16) (Figures 4a–4d), and AUC was 0.9560 (Figure 4e).

## Discussion

As gastric IM is widely accepted as a precancerous lesion, its correct diagnosis and long-term follow up of subjects are important. Although the pathological criteria for IM in the Updated Sydney System criteria are regarded as a gold standard for its diagnosis, the accuracy of endoscopy for IM identification remains poor when using these criteria.

Kaminishi et al. found that ash-colored nodular changes were specific (98–99%), but not sensitive (6–12%), for identifying histological intestinal metaplasia, and concluded that conventional endoscopy is unsuitable for diagnosing gastritis with IM [22]. Many studies have investigated using ME to overcome the limitations of diagnosing IM with conventional endoscopy [3], [10]. ME with methylene blue staining can help diagnosing IM [23], but requires tedious preparation (including the use of mucolytic agents, spraying dye, and irrigating of the mucosal surface) and carries the risk of oxidative DNA damage [24]. NBI is easy to use, and offers chromoendoscopy with no need for additional dye spray.

As NBI-ME has been shown to reveal mucosal details, which may increase IM detection, the accuracy of LBC-based diagnosis of IM has been focus of several studies [3], [10], [11], [17]. Our meta-analysis shows that pooled sensitivity and specificity of LBC are high in diagnosing gastric IM, no matter how many studies it includes; the higher AUC seen in the 3-study analysis indicates even better accuracy. The LBC sign could improve both diagnostic accuracy and biopsy targeting for gastric IM under endoscopy.

Management of gastric IM includes endoscopic surveillance, *H. pylori* eradication and chemoprevention, and endoscopic surveillance plays a key role [25]. One study included in our meta-analysis followed 26/38 patients at 1-year intervals, and found that LBC under NBI-ME can be used effectively to surveil gastric IM, but did not find the optimal duration of follow-up [17]. The first European Guidelines on management of precancerous conditions and lesions in the stomach recommended that patients with extensive atrophic gastritis (AG) and/or extensive IM should be offered endoscopic surveillance every 3 years [25]. However, Zullo A. et al proposed that follow-up should be individualized, and suggested aggressive (1 year) follow-up in patients with risk factors and less intensive (2–3 years) follow-up in other patients [26]. Patient-tailored endoscopic surveillance may be more appropriate than a single schedule for all patients.

Our meta-analysis show that LBC sign is accurate in diagnosing IM in gastric mucosa. However, its diagnostic value in Barrett's esophagus (BE) is unclear. One study showed LBC under NBI-ME to be 79% sensitive, 97% specific, and 89% accurate in diagnosing IM in BE [27]. The diagnostic yield of LBC in BE deserve further study.

There are some deficiencies in diagnosing of IM under NBI-ME in our study. First, no consensus on the diagnostic criteria of IM with LBC has been established. Of 9 references [3], [10], [11], [14]–[17], [19], [21] about diagnosing gastric IM using LBC under NBI endoscopy, 7 references [3], [10], [11], [15], [17], [19], [21] used “with or without LBC” as the standard criterion in this consideration. Different criteria were also used in two studies. In one study, LBC>10% at each field was used to define positive LBC, and was 72.1% sensitive, 96% specific, and 84.5% accurate, in diagnosing IM by endoscopy [14]. In another study, LBC combined with irregular mucosa was used to diagnose IM, and was 71% sensitive and 58% specific [16]. The definition of positive LBC and morphological changes in the mucosa and microvessels may influence the diagnostic sensitivity and specificity of intestinal metaplasia. Thus, LBC as a diagnostic criterion for IM requires for further large-sample studies.

Although the diagnostic criteria and patient selection were strictly defined in the included studies, they showed obvious heterogeneity. One study from Thailand using ROC analysis showed evident bias [17]. The same situation occurred in another study that included a per-patient analysis, with sensitivity of 0.80 (95%CI: 0.67–0.92) and specificity of 0.96 (95%CI: 0.93–0.99) [21]; its sensitivity was better than the Thai study, but lower than the other three studies [3], [10], [11]. Statistically, per-patient sensitivity should be better than per-lesion sensitivity. In three [3], [10], [11] of the five articles, the biopsy protocols are similar, biopsy samples were taken from each area showing LBC and from adjacent non-LBC mucosa in LBC^+^ patients. However, biopsy samples were not taken from adjacent non-LBC mucosa in the other two articles [17], [21]. The bias of these two studies compared with the other three studies might be attributed to differences in endoscopy types (Olympus GIF−Q240Z/260Z vs Olympus GIF−Q160Z), biopsy protocols, race of patient cohort (East Asians vs other), and other factors (such as proficiency of physicians). Because the number of studies of endoscopy types, subjects' race and clinicians' proficiency was limited, subgroup analysis for accuracy of LBC-based diagnosis of IM could not be performed. After eliminating the study from Thailand, the pooled sensitivity and specificity were favorable.

The present meta-analysis has demonstrated that LBC under NBI-ME to be highly sensitive and specific in diagnosing gastric IM. This method can increase the accuracy of endoscopy for gastric intestinal metaplasia, guide endoscopic biopsy, and increase positive findings on pathological examination.

## Supporting Information

Checklist S1 (DOC)
